# A CRISPR-Cas12a-based diagnostic method for multiple genotypes of severe fever with thrombocytopenia syndrome virus

**DOI:** 10.1371/journal.pntd.0010666

**Published:** 2022-08-02

**Authors:** Bum Ju Park, Jeong Rae Yoo, Sang Taek Heo, Misun Kim, Keun Hwa Lee, Yoon-Jae Song

**Affiliations:** 1 Department of Life Science, Gachon University, Seongnam-Si, Korea; 2 Division of Infectious Diseases, Jeju National University School of Medicine, Jeju, South Korea; 3 Department of Microbiology, Hanyang University College of Medicine, Seoul, South Korea; NIAID Integrated Research Facility, UNITED STATES

## Abstract

Severe fever with thrombocytopenia syndrome virus (SFTSV) infection is commonly reported in countries of Northeast Asia including China, Japan and South Korea. The majority of the SFTS patients are elderly and the average fatality rate is more than 10%. A rapid and sensitive diagnostic method to monitor and prevent SFTSV transmission remains an urgent clinical challenge. In this study, we developed a molecular diagnostic technique for detection of SFTSV using the CRISPR-Cas12a system combined with reverse transcription recombinase polymerase amplification (RT-RPA). Using this method, we successfully diagnosed SFTSV infections with the reaction time of 50 min from blood plasma without cross-reactivity to other viruses, supporting its application for rapid and sensitive diagnosis of SFTS.

## Author summary

Severe fever with thrombocytopenia syndrome (SFTS) is an emerging infectious disease caused by *Dabie bandavirus*, also known as SFTSV, and reported in countries of Northeast Asia including China, Japan and South Korea. The average fatality rate is more than 10% and reported to increase with age. Currently, no vaccines or targeted treatments for SFTS are available and only symptomatic therapy is offered. Thus, SFTSV diagnosis at early stages is critical for successful therapeutic outcomes. In this study, we developed a molecular diagnostic technique for detection of SFTSV using the CRISPR-Cas12a system combined with reverse transcription recombinase polymerase amplification (RT-RPA). Using this method, we successfully diagnosed SFTSV infections under isothermal conditions with the reaction time of 50 min from blood plasma.

## Introduction

Severe fever with thrombocytopenia syndrome (SFTS) is an emerging infectious disease caused by *Dabie bandavirus*, also known as SFTSV. The first suspected cases were initially reported in rural areas of Hubei and Henan provinces in China in 2009 and the virus was isolated in 2011 [1]. Since 2013, SFTS has been confirmed in Japan and south Korea, with continued reports of infections in these countries [2,3]. SFTSV is transmitted by tick bites from *Haemaphysalis longicornis* native to China, Japan and South Korea that is also a well-known invasive species in Australia, New Zealand and the Pacific islands [4]. Six genotypes of SFTSV have been classified (A, B, C, D, E and F) based on large segment (L), medium segment (M) and small segment (S) sequences. In China, all six genotypes are prevalent, while in South Korea, genotype B accounts for the majority of cases, along with a lower incidence of A, D and F genotypes [5].

SFTSV is a single-stranded negative RNA consisting of three segments: L, M and S. The L segment encodes RNA-dependent RNA polymerase that functions in replication and transcription of viral RNA. The M segment encodes a glycoprotein precursor, which is processed to glycoprotein N (Gn) and C (Gc) constituting the envelope. The S segment, an ambisense RNA, encodes two proteins. The antisense RNA encodes nucleocapsid protein (NP) and sense RNA encodes nonstructural proteins (NS). NP functions in viral replication and transcription while NS regulates host immune responses [1,6,7].

High fever, muscle pain, headache, abdominal pain, fatigue, vomiting and coughing and clinical symptoms, including thrombocytopenia, leukopenia and gastrointestinal bleeding, are common indications of SFTS infection [1,8]. The mortality rates of SFTS in China, Japan and Korea are 4.8%, 27% and 23.3%, respectively, and reported to increase with age [6,9]. On average, elderly individuals over 60 years of age are more susceptible to infection [10–12]. Currently, no vaccines or targeted treatments for SFTS are available and only symptomatic therapy is offered, highlighting the importance of SFTSV diagnosis at early stages for successful therapeutic outcomes.

Nucleic acid-based tests (NAT) using quantitative reverse-transcription polymerase chain reaction (qRT-PCR) or RT-PCR are considered as the best known technology for SFTSV diagnosis. While these methods can effectively detect viruses with low copy numbers and various SFTSV subtypes, application in the field is difficult, since access to a thermocycler, RNA extraction process and skilled laboratory technicians are required for diagnosis. An enzyme-linked immunosorbent assay (ELISA) to detect antibodies against SFTSV has additionally been developed for this purpose, although not on a commercial basis [13].

Recently, a field-applicable rapid molecular diagnostic technique using clustered, regularly interspaced, short palindromic repeats (CRISPR) and CRISPR-associated (Cas) system was introduced [14,15]. Cas12a from *Lachnospiraceae* bacterium (LbCas12a) can recognize target DNA using guide RNA (gRNA) complementary to the target DNA sequence with the T nucleotide-rich protospacer-adjacent motif (PAM) sequence [16–20]. After recognition, LbCas12a is activated and induces non-specific single-strand DNA cleavage [21]. Based on the trans-cleavage activity of LbCas12a and isothermal nucleic acid amplification, a novel molecular diagnostic method designated ‘DNA endonuclease-targeted CRISPR trans reporter’ (DETECTR) has been developed [21] and successfully applied in the diagnosis of viral infections including severe acute respiratory syndrome coronavirus 2 (SARS-CoV-2), human papilloma viruses (HPV), and influenza A and B viruses (IAV and IBV) [21–23]. In this study, we developed a rapid, sensitive and field-applicable diagnostic method for detecting SFTSV by grafting DETECTR to heating unextracted diagnostic samples to obliterate nucleases (HUDSON), which degrades virus particles. Results were evaluated using the lateral flow strip.

## Materials and methods

### Ethics statement

This study was approved by the Institutional Review Board (IRB) of Jeju National University Hospital (IRB file no. 2018-11-002). Written informed consent was obtained from all the participants.

### Viruses

Propagation and titration of influenza A virus (IAV) (A/Puerto Rico/8/1934), influenza B virus (IBV) (B/Brisbane/60/2008), hepatitis C virus (HCV) (JFH-1) and hepatitis E virus (HEV) (47832c) were performed as described previously [24–27]. To produce lentivirus pseudotyped with SFTSV S gene fragment, pLenti CMV SFTSV S gene fragment vectors were generated via GATEWAY cloning using pLenti CMV Puro DEST. The pLenti CMV Puro DEST (w118-1) construct was a gift from Eric Campeau and Paul Kaufman (Addgene #17452; http://n2t.net/addgene:17452; RRID: Addgene 17452) [28]. The S gene fragment (nucleotides 1216 to 1370) of SFTSV genotype B (GenBank accession number: KP663745) [5] was synthesized (Macrogen, Seoul, Korea) and amplified via PCR using the following primers: forward (5’-GGGGACAAGTTTGTACAAAAAAGCAGGCTAAAAGTCTGAGCCTTCGCTTCT -3’) and reverse (5’-GGGGACCACTTTGTACAAGAAAGCTGGCTATGTTCTTCTCCATCAAGAAC-3’). Subsequently, BP and LR Gateway reactions (Invitrogen, Waltham, MA, USA) were performed to generate the pLenti CMV SFTS S gene segment.

For production of lentivirus pseudotyped with SFTSV S gene, HEK293T cells were co-transfected with pLenti CMV SFTSV S gene segment, pMD2.G, pMDLg/pRRE and pRSV-Rev using Omicsfect according to the manufacturer’s instructions (Omics Biotechnology, Seoul, Korea). pMD2.G, pMDLg/pRRE and pRSV-Rev were gifts from Didier Trono (pMD2.G Addgene plasmid # 12259; http://n2t.net addgene:12259; RRID:Addgene_12259, pMDLg/pRRE Addgene plasmid # 12251; http://n2t.net/addgene:12251; RRID:Addgene_12251, pRSV-Rev Addgene plasmid # 12253; http://n2t.net/addgene:12253; RRID:Addgene_12253)) [29]. At 48 h after transfection, the culture medium was passed through a 0.45μm filter (Sartorius, Göttingen, Germany) to obtain virus. Prior to transduction, polybrene was added to the filtrate at a final concentration of 10 μg/mL. Lentiviral titer was determined according to the Limiting Dilution-Colony Counting protocol at MD Anderson Cancer Center (https://www.mdanderson.org/). Handling of viruses was performed in a Biosafety level2 (BSL-2) laboratory.

### SFTSV RNA synthesis

SFTSV RNAs were synthesized by *in vitro* transcription (IVT) reaction and used to determine the sensitivity and specificity of SFTSV DETECTR. The S gene fragments (nucleotides 1216 to 1370) of SFTSV genotypes A, B, C, D, E and F (GenBank accession numbers: KF791948, KP663745, AB985541, KP 663733, HQ141606 and KF358693, respectively) [5] were synthesized (Macrogen, Seoul, Korea) and amplified by PCR using primers containing a T7 promoter (S1 Table). IVT reaction was carried out with the PCR products by using the mMESSAGE mMACHINE T7 Transcription kit (Invitrogen, Waltham, MA, *USA*) according to the manufacturer’s instructions. The synthesized SFTSV RNAs were purified using RNA Clean and Concentrator 5 columns (Zymo research, Irvine, CA, USA).

### Clinical samples

Among the 9 patients tested, 4 SFTS cases were laboratory-confirmed at Jeju National University Hospital (Jeju, South Korea). For molecular diagnosis of SFTSV, RNA was extracted from stored patient serum using a QIAamp Viral RNA Mini Kit (QIAGEN, Hilden, Germany). RT-PCR was performed to amplify the small (S) segment of viral RNA from stored serum as reported previously [30, 31].

### HUDSON

Viruses were lysed using Heating Unextracted Diagnostic Samples to Obliterate Nucleases (HUDSON) as described previously [32]. For inactivation of RNase in culture media of IAV, IBV, HCV and HEV, TCEP and EDTA were added to samples at final concentrations of 100 mM and 1 mM, respectively. The mixtures were incubated in a thermocycler at 50°C for 20 min, followed by 95°C for 5 min. For RNase inactivation in blood plasma, TCEP and EDTA were added to samples at final concentrations of 100 mM and 1 mM, respectively. Samples were incubated at 50°C for 5 min, followed by 64°C for 5 min using a thermocycler. RNase-free water was added at a ratio of 1:4 to prevent solidification of the mixture after HUDSON.

### DETECTR

Viral RNA was amplified via RT-RPA using the TwistAmp Basic kit (TwistDx, Cambridge, UK) with primers designed according to the manufacturer’s manual (Table 1, S1 Fig) [23]. The RT-RPA reaction mixture contained 29.5 μL rehydration buffer, 2.4 μL forward and reverse primers (10 μM each), 1 μL SuperScript IV Reverse Transcriptase (Invitrogen, Waltham, *Massachusetts*, USA), 1 μL RNase inhibitor (Enzynomics, Daejeon, Korea) and 2.5 μL of 280 mM magnesium acetate. Samples and nuclease-free water were added to the reaction mixture to obtain a final volume of 50 μL. In the case of HUDSON-treated virus-containing culture medium, 1 μL of sample was added to the reaction mixture while for HUDSON-treated patient plasma, 5 μL of sample was used. Reaction mixtures were incubated at 42°C for 30 min.

**Table 1:** RPA primers and gRNAs for SFTSV DETECTR.

		Virus	Gene	Sequence	PAM
RT-RPA primers	SFTS-S-F	SFTSV	S	AAGTCTGAGCCTTCGCTTCTCTATGGCTTCAAGAG	
	SFTS-S-R	SFTSV	S	TGTTCTTCTCCATCAAGAACAGCTGGGCAATGGAA	
gRNAs	S1	SFTSV	S	AAGGGGACATGATATTGGAT	TTTG
	S2	SFTSV	S	TTATCCTGTGGAAGAGGCCC	TTTG

LbCas12a trans-cleavage assays were performed as described previously [21–23]. To design gRNAs corresponding to all SFTSV genotypes, the previously reported SFTSV variant sequences were screened for the PAM sequence (TTTV, V is a A, C or G) (Table 1, S1 Fig). Two gRNAs corresponding to SFTSV S gene (S1 and S2 which correspond to nucleotides 1252 to 1271 and 1296 to 1315, respectively) were designed and synthesized to detect all SFTSV genotypes (Bioneer, Daejeon, Korea). To generate the LbCas12a-gRNA complex, LbCas12a and gRNA were added to 1× NEBuffer 2.1 to obtain final concentrations of 50 nM and 62.5 nM, respectively. The reaction mixture was incubated at 37°C for 30 min for the RT-RPA reaction. For fluorescence assays, 2 μL of RT-RPA products, 80 μL of 1× NEBuffer 2.1, 18 μL of LbCas12a-gRNA complex and 2 μL of 10 μM FQ-labeled reporter (/56-FAM/TTATT/3IABkFQ/; Integrated DNA Technologies, Coralville, IA, USA) were added directly to 96-well microplates. Fluorescence results were obtained after incubation at 37°C for 10 min (λex, 485 nm; λem, 535 nm). For the lateral flow assay, RT-RPA products (2 μL) were incubated with 40 μL of 1× NEBuffer 2.1, 36 μL of LbCas12a-gRNA complex and 2 μL of 10 μM lateral flow cleavage reporter (/56-FAM/TTATT/3Bio/; Integrated DNA Technologies) at 37°C for 10 min. Milenia HybriDetect 1 lateral flow strips (Milenia, Giesesen, Germany) was applied to the incubated samples according to the manufacturer’s instructions, and results determined after 2 min [22].

## Results

### Detection of SFTSV using DETECTR

**Fig 1:**
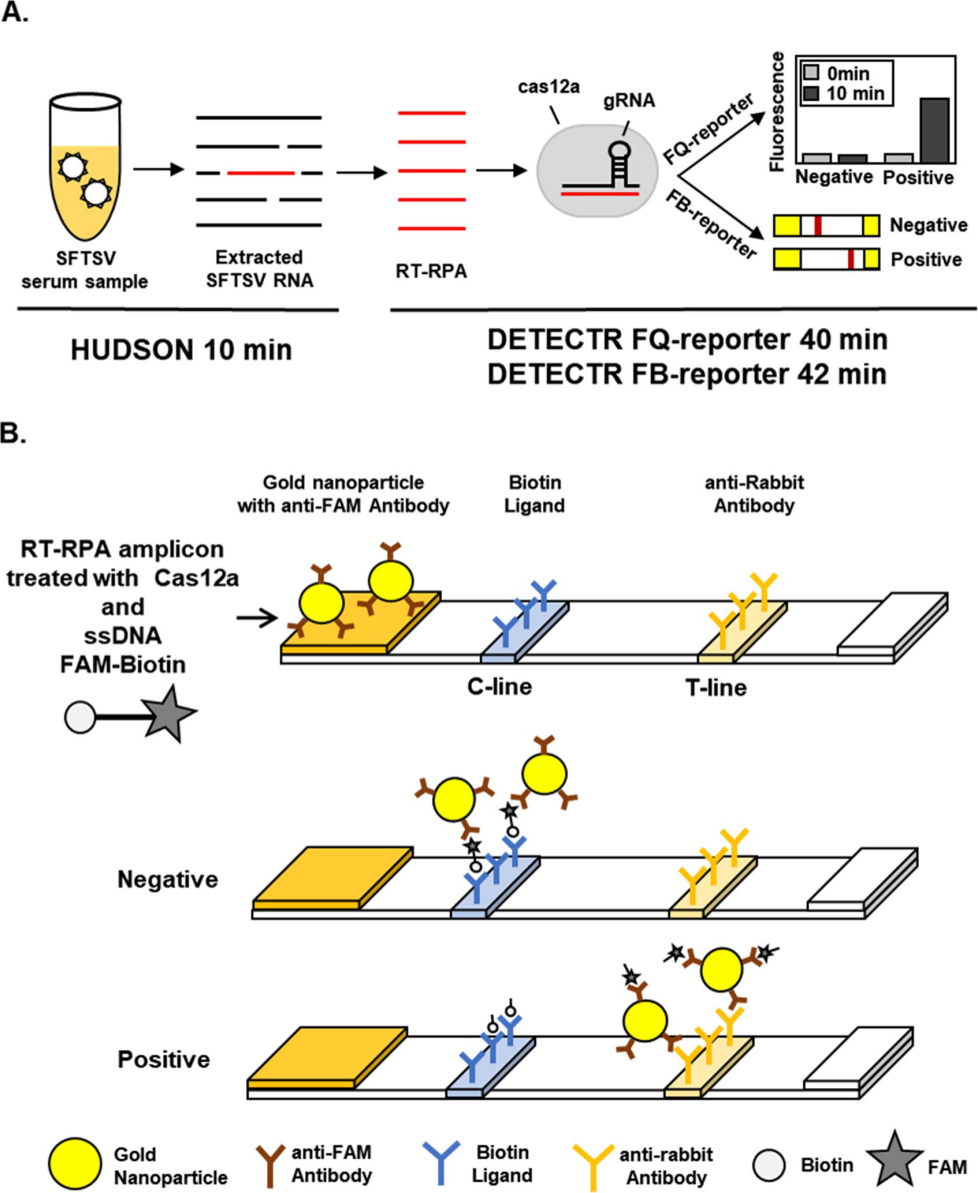
SFTSV DETECTR. (A) Graphical workflow of SFTSV DETECTR. (B) Schematic presentation of positive and negative results obtained using a lateral flow strip. In a positive result, a band may appear at the C-line possibly due to the partial cleavage of the FB-reporter by Cas12a. FQ-reporter, fluorescence-quencher reporter; FB-reporter, FAM-Biotin lateral flow strip reporter; C-line, control line; T-line, test line.

**Fig 2:**
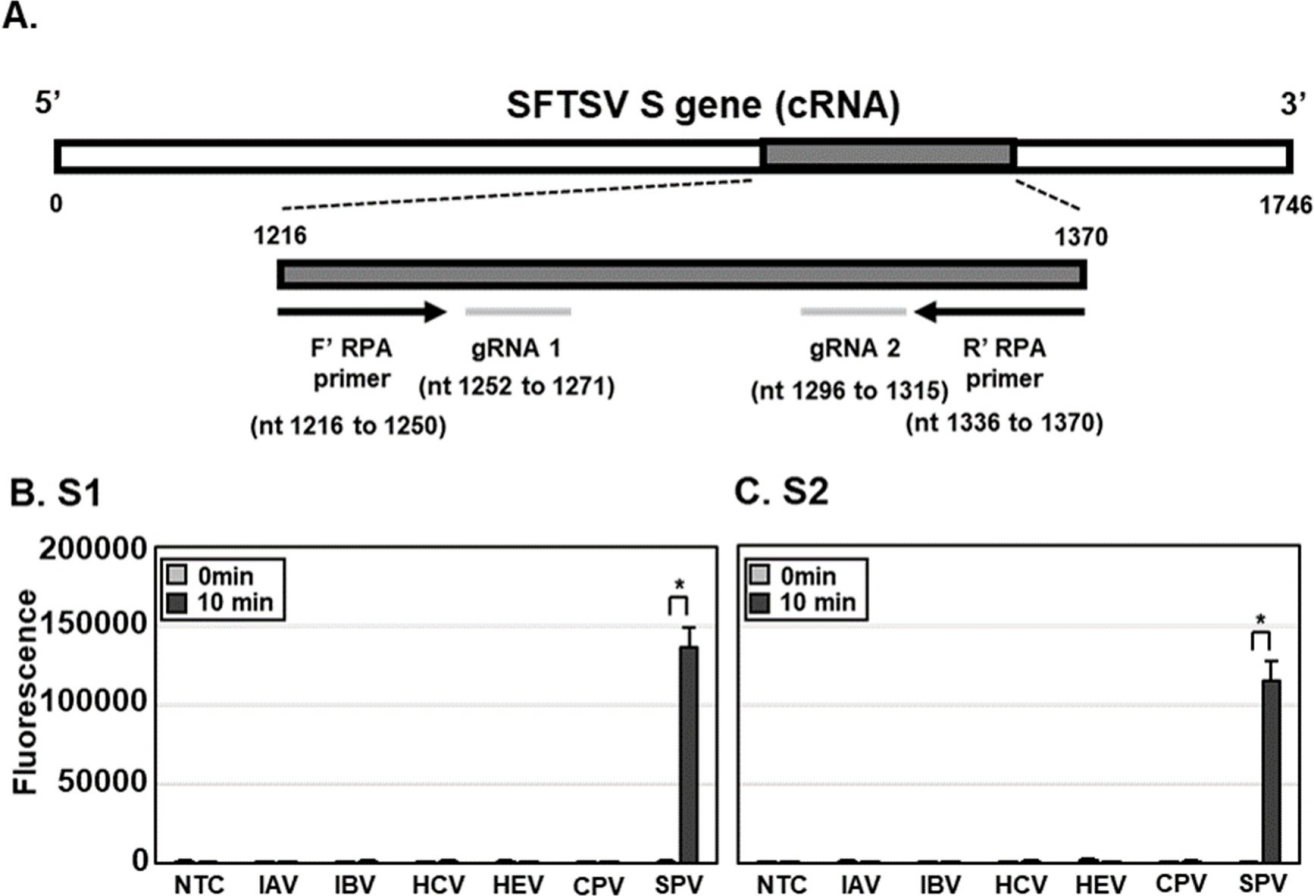
Detection of lentiviruses pseudotyped with SFTSV S gene using DETECTR combined with fluorescence assay. (A) A diagram of gRNAs and primer sets for SFTSV DETECTR. (B and C) Lentiviruses pseudotyped with and without SFTSV S gene (SPV and CPV, respectively) (100 CFU), IAV and IBV (100 PFU) and HCV and HEV (100 genome copies) were lysed via HUDSON and viral nucleic acids amplified via RT-RPA with a primer set specific for the SFTSV S gene. RT-RPA amplicons were detected with SFTSV DETECTR combined with fluorescence assay using gRNAs (A) S1 and (B) S2 corresponding to the S genes of all SFTSV genotypes. Fluorescence saturation occurred within 10 min. Values are presented as means ± s.d. (error bars) (*n* = 3 replicates; * *p* < 0.05 between samples, two-sample *t*-test). *nt*, *nucleotides;* PFU, plaque forming unit; CFU, colony forming unit; SPV, SFTSV pseudovirus; CPV, control pseudovirus.

DETECTR for SFTSV (SFTSV DETECTR) was designed to facilitate rapid and sensitive diagnosis (Fig 1). Diagnostic samples containing SFTSV were lysed via HUDSON and viral nucleic acids amplified using reverse transcription recombinase polymerase amplification (RT-RPA) with primer sets specific for the SFTSV S gene (Fig 2A, Table 1). RT-RPA amplicons were incubated with LbCas12a and gRNA targeting SFTSV S gene and the trans-cleavage activity of LbCas12a determined with a fluorescence assay using a ssDNA-fluorophore (FAM) quencher (FQ)-labeled reporter or lateral flow assay (LFA) using a ssDNA-FAM-Biotin reporter (Fig 1).

To test and optimize SFTSV DETECTR, lentivirus pseudotyped with the SFTSV S gene segment was generated. Unextracted lentiviruses pseudotyped with SFTSV S gene segment were lysed through HUDSON, followed by application of SFTSV DETECTR combined with a fluorescence assay. IAV, IBV, HCV and HEV were used as controls to establish the specificity of SFTSV DETECTR. Both gRNAs successfully targeted RT-RPA amplicons. Moreover, SFTSV DETECTR combined with the fluorescence assay successfully detected lentivirus pseudotyped with SFTSV S gene without cross-reacting with IAV, IBV, HCV and HEV (Fig 2B and 2C). The fluorescence signal started to be saturated after 10 min of incubation (S2 Fig). The minimum reaction time required for HUDSON, RT-RPA and target detection using fluorescence were 10, 30 and 10 min, respectively (Fig 1A). The lateral flow assay required an additional 2 min for result interpretation.

To further determine whether SFTSV DETECTR can detect all SFTSV genotypes, the *in vitro*-transcribed RNA fragments of S gene from SFTSV genotypes A, B, C, D, E and F were generated. SFTSV DETECTR combined with the fluorescence assay successfully detected the *in vitro*-transcribed RNA fragments of S gene from all SFTSV genotypes (S3 Fig) Thus, our SFTSV DETECTR system could effectively identify all SFTSV genotypes with the reaction time of 50 and 52 min based on fluorescence and LFA, respectively.

### Determination of limit of detection (LoD) of SFTSV DETECTR

**Fig 3:**
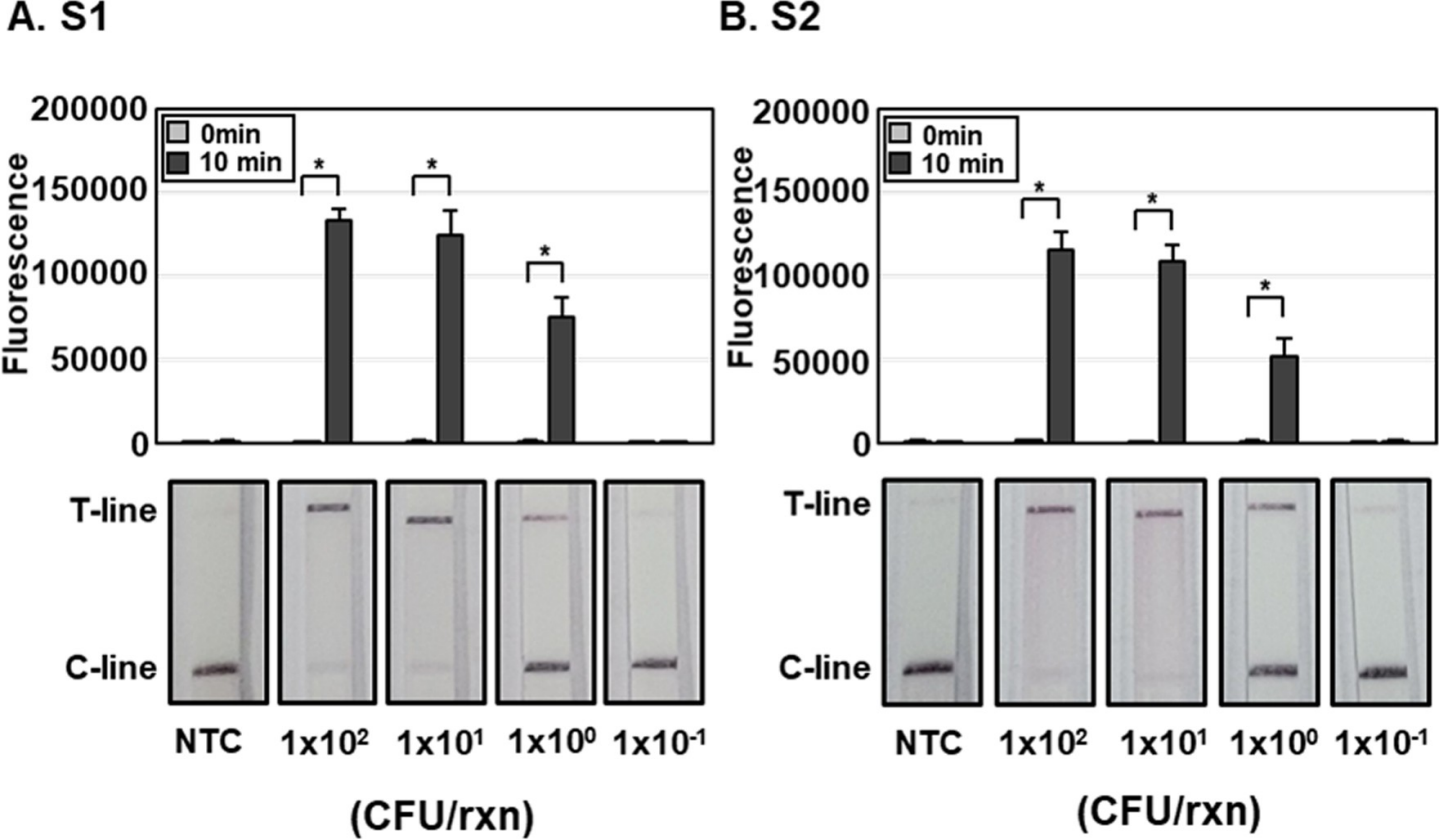
Sensitivity of SFTSV DETECTR. Different concentration of lentiviruses pseudotyped with SFTSV S gene (1.0 × 10^−1^ to 1.0 × 10^2^ CFU per reaction) were lysed through HUDSON and viral nucleic acids amplified via RT-RPA with a primer set specific for the SFTSV S gene. RT-RPA amplicons were detected using SFTSV DETECTR combined with fluorescence assay or lateral flow using gRNAs (A) S1 and (B) S2 corresponding to the S genes of all SFTSV genotypes. Fluorescence saturation occurred within 10 min. Lateral flow assay results were evaluated 2 min after the strip was reacted with the sample. Values are presented as means ± s.d. (error bars) (*n* = 3 replicates; * *p* < 0.05 between samples, two-sample *t*-test). C-line, control line; T-line, test line; NTC, no template control. CFU, colony forming unit.

Lentiviruses pseudotyped with SFTSV S gene at various CFU per reaction were lysed with HUDSON and SFTSV DETECTR performed to detect SFTSV (Fig 3). Using both fluorescence and LFA, SFTSV DETECTR was able to detect lentiviruses pseudotyped with the SFTSV S gene up to 1 x 10^0^ CFU per reaction (Fig 3). The LoD of SFTSV DETECTR was further determined using the *in-vitro* transcribed RNA fragments of SFTSV S gene. Using both fluorescence and LFA, SFTSV DETECTR was able to detect SFTSV RNA fragments up to 1 x 10^2^ copies per reaction (S4 Fig). Both S1 and S2 gRNAs showed similar activity in detection of SFTSV (Figs 3 and S4).

### Application of SFTSV DETECTR to clinical samples

**Fig 4:**
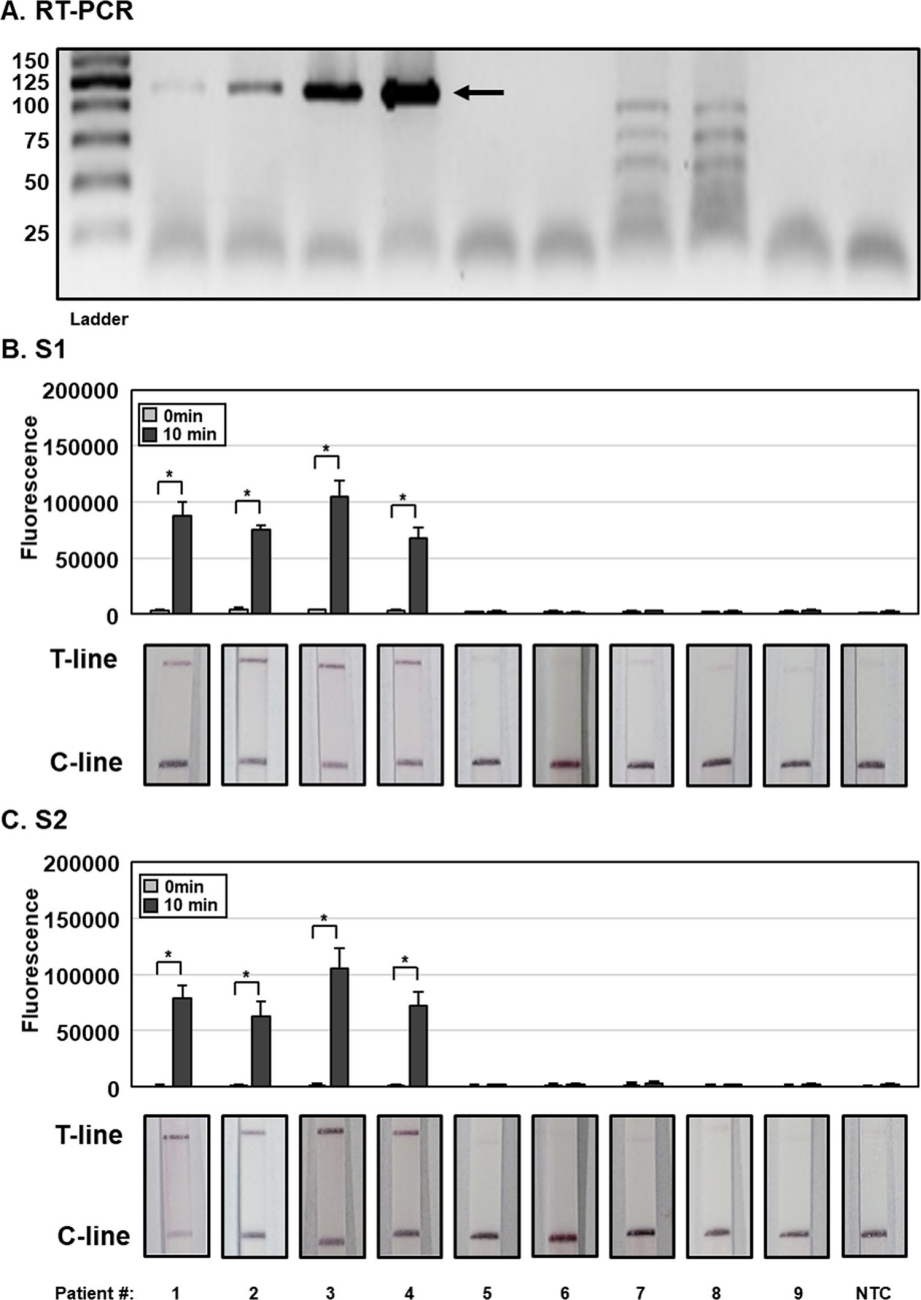
Application of SFTSV DETECTR to clinical samples. (A) RNA was extracted from patient plasma samples and subjected to RT-PCR for SFTSV detection. PCR products (124 bp) were visualized using gel electrophoresis. (B, C) HUDSON-treated patient plasma samples were used to amplify viral nucleic acids using RT-RPA with a primer set specific for the SFTSV S gene. RT-RPA amplicons were detected using SFTSV DETECTR combined with fluorescence assay or lateral flow using gRNAs (A) S1 and (B) S2 corresponding to the S genes of all SFTSV genotypes. Fluorescence saturation occurred within 10 min. Lateral flow assay results were evaluated 2 min after the strip was reacted with the sample. Values are presented as means ± s.d. (error bars) (*n* = 3 replicates; * *p* < 0.05 between samples, two-sample *t*-test). C-line, control line; T-line, test line; NTC, no template control.

To evaluate clinical applicability of SFTSV DETECTR, a pilot study to detect SFTSV in patient plasma samples was undertaken, and results were compared with RT-PCR diagnostic test. Among 9 patients hospitalized with suspected SFTSV infection, 4 (Patients # 1–4) were confirmed as infected with SFTSV based on RT-PCR (Fig 4A). Consistent with RT-PCR results, SFTSV DETECTR combined with the fluorescence assay and LFA using S1 and S2 gRNAs confirmed infection in the same 4 patients (Patients # 1–4) (Fig 4B and 4C). Our findings clearly indicate that SFTSV DETECTR has comparable sensitivity to RT-PCR for SFTSV detection in clinical samples.

## Discussion

CRISPR-Cas12a, like cas9, was first reported as a tool for genome editing [20]. Since then, Cas12a has been applied in various fields such as agriculture and plant biotechnology, gene therapy and molecular diagnostics [33,34]. In this study, we developed a rapid and sensitive CRISPR-Cas12-based diagnostic method for SFTSV (SFTSV DETECTR), which could successfully detect SFTSV at 1 x 10^2^ viral RNA copies per reaction with the reaction time of 50 and 52 min in combination with fluorescence and LFA, respectively. Two gRNAs were designed and utilized in SFTSV DETECTR for detection of all reported SFTSV genotypes [5,31]. RPA was selected for isothermal amplification of viral RNA. In the case of LAMP, a well-known isothermal amplification method, 4 or more primers are required. However, in this case, amplification of various genotypes with one set of LAMP primers was difficult and this technique was therefore not adopted. The SFTSV DETECTR system exhibited no cross-reactivity for other RNA viruses including IAV, IBV, HCV and HEV.

Although the LoD of SFTSV DETECTR was higher than that of RT-PCR (2 RNA genome copies per reaction) [35], it displayed comparable sensitivity to RT-PCR for SFTSV detection in clinical samples (Fig 4). SFTSV DETECTR required less time than conventional diagnostic methods for virus detection in plasma samples of patient. Without performing RNA extraction, viral nucleic acids from plasma samples of patients were successfully lysed via HUDSON and amplified and detected with SFTSV DETECTR under isothermal conditions with the reaction time of 50 min. Our results confirmed the clinical applicability of SFTSV DETECTR, supporting its potential as an alternative for RT-PCR in the future. Clinical validation of SFTSV DETECTR with a large number of SFTS patient samples is the subject of future studies.

To ensure field-deployability, a lateral flow strip was adopted for rapid assessment of the readouts. LFA results were as sensitive and accurate as those obtained with the fluorescence assay. One drawback of using LFA was the development of a positive band after 2 min of reaction. In addition, we conducted RT-RPA at 37°C (not 42°C) or target detection assay at 42°C (not 37°C) to synchronize reaction temperatures. Although the fluorescence signal was reduced by 25%, reading the results did not pose a problem. Currently, SFTSV cases have been also reported in low and low middle income countries (LMIC) with the lack of public health infrastructure or budget (e.g., Southeast Asia), so a simple and inexpensive method such as a heating block and a LFA will be preferred rather than a method using expensive equipment such as a thermocycler. Since the remaining solutions except for the RNA sample can be made and used in the form of a master mix, SFTSV DETECTR can be applied to develop a self-diagnostic test kit which can be used to people in rural and remote areas with difficult access to health care. A SFTSV DETECTR kit requires only a heating block and a lateral flow strip for LFA. Thus SFTSV DETECTR is expected to be more suitable for in-field diagnostics compared to existing PCR-based tests. With the development of novel sample pre-treatment methods that do not require the use of thermocycler and troubleshooting methods for false-positive bands of LFA, SFTSV DETECTR could be effectively used to establish a field-deployable diagnostic test kit.

## Supporting information

S1 Fig Alignment of RPA primers and gRNAs with the target regions of SFTSV genotypes. Nucleic acid sequences of (A) RPA primers and (B) gRNAs used in this study are aligned with S gene sequences of SFTSV genotypes A, B, C, D, E and F. Nucleic acid sequences of SFTS-S-R and S1 gRNA are shown in the reverse-complement orientation.
(TIF)

S2 Fig The fluorescence intensities of SFTSV DETECTR with gRNAs S1 and S2 over the time course. LbCas12a trans-cleavage assays were performed with 10^2^ copies of *in vitro*-transcribed SFTSV RNA fragments, and fluorescence signals were determined at every 5 min over the course of 30 min. NTC, no template control.
(TIF)

S3 Fig Detection of SFTSV genotypes by SFTSV DETECTR. The *in vitro*-transcribed RNA fragments of S gene from SFTSV genotypes A, B, C, D, E and F were amplified via RT-RPA with a primer set specific for the SFTSV S gene. RT-RPA amplicons were detected using SFTSV DETECTR combined with fluorescence assay or lateral flow using gRNAs (A) S1 and (B) S2 corresponding to the S genes of all SFTSV genotypes. Fluorescence saturation occurred within 10 min. Lateral flow assay results were evaluated 2 min after the strip was reacted with the sample. Values are presented as means ± s.d. (error bars) (*n* = 3 replicates; * *p* < 0.05 between samples, two-samples t-test). NTC, no template control.
(TIF)

S4 Fig Determination of LoD of SFTSV DETECTR. Different copy numbers of *in vitro*-transcribed RNA fragments of SFTSV S gene were amplified via RT-RPA with a primer set specific for the SFTSV S gene. RT-RPA amplicons were detected using SFTSV DETECTR combined with fluorescence assay or lateral flow using gRNAs (A) S1 and (B) S2 corresponding to the S genes of all SFTSV genotypes. The fluorescence was measured at 10 min. Lateral flow assay results were evaluated 2 min after the strip was reacted with the sample. Values are presented as means ± s.d. (error bars) (*n* = 3 replicates). C-line, control line; T-line, test line.
(TIF)

S1 Table PCR primers for IVT. (DOCX)
