# Supplementary figures and images for: A CRISPR-Cas12a-based diagnostic method for multiple genotypes of severe fever with thrombocytopenia syndrome virus
Source: PLoS Negl Trop Dis. 2022 Aug 2;16(8):e0010666. doi: 10.1371/journal.pntd.0010666 (PMC9345333; doi:10.1371/journal.pntd.0010666)

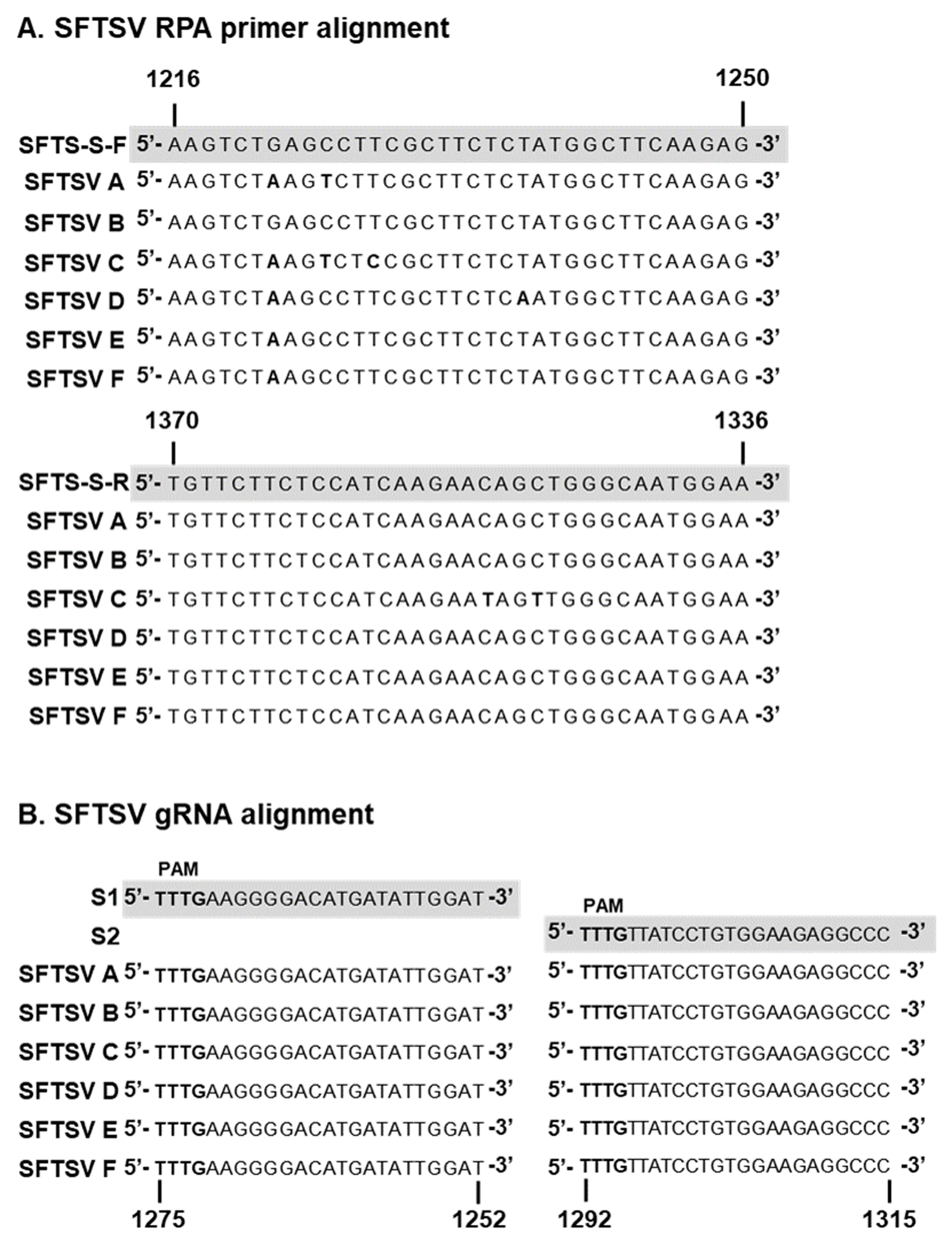

Supplement: S1 Fig — Nucleic acid sequences of (A) RPA primers and (B) gRNAs used in this study are aligned with S gene sequences of SFTSV genotypes A, B, C, D, E and F. Nucleic acid sequences of SFTS-S-R and S1 gRNA are shown in the reverse-complement orientation. (TIF) [file pntd.0010666.s001.tif]

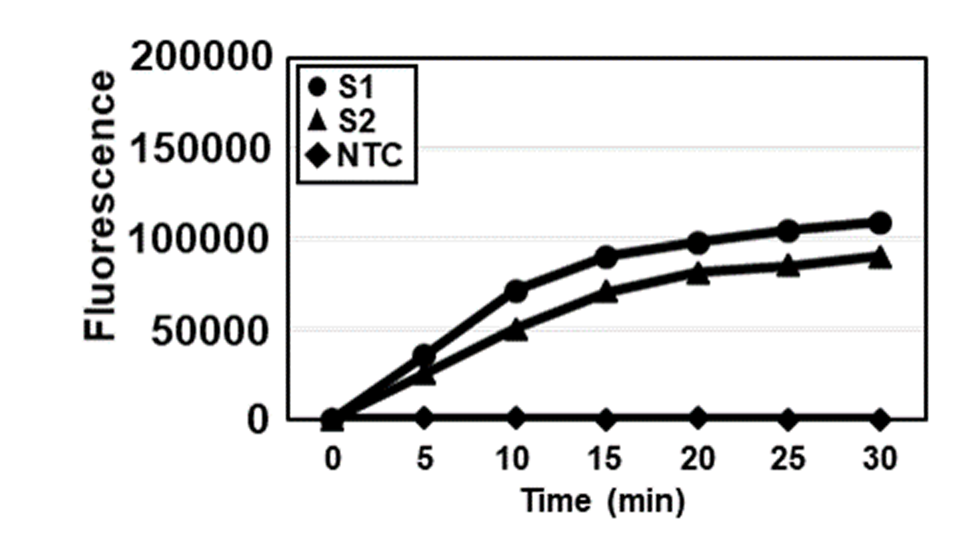

Supplement: S2 Fig — LbCas12a trans-cleavage assays were performed with 102 copies of in vitro-transcribed SFTSV RNA fragments, and fluorescence signals were determined at every 5 min over the course of 30 min. NTC, no template control. (TIF) [file pntd.0010666.s002.tif]

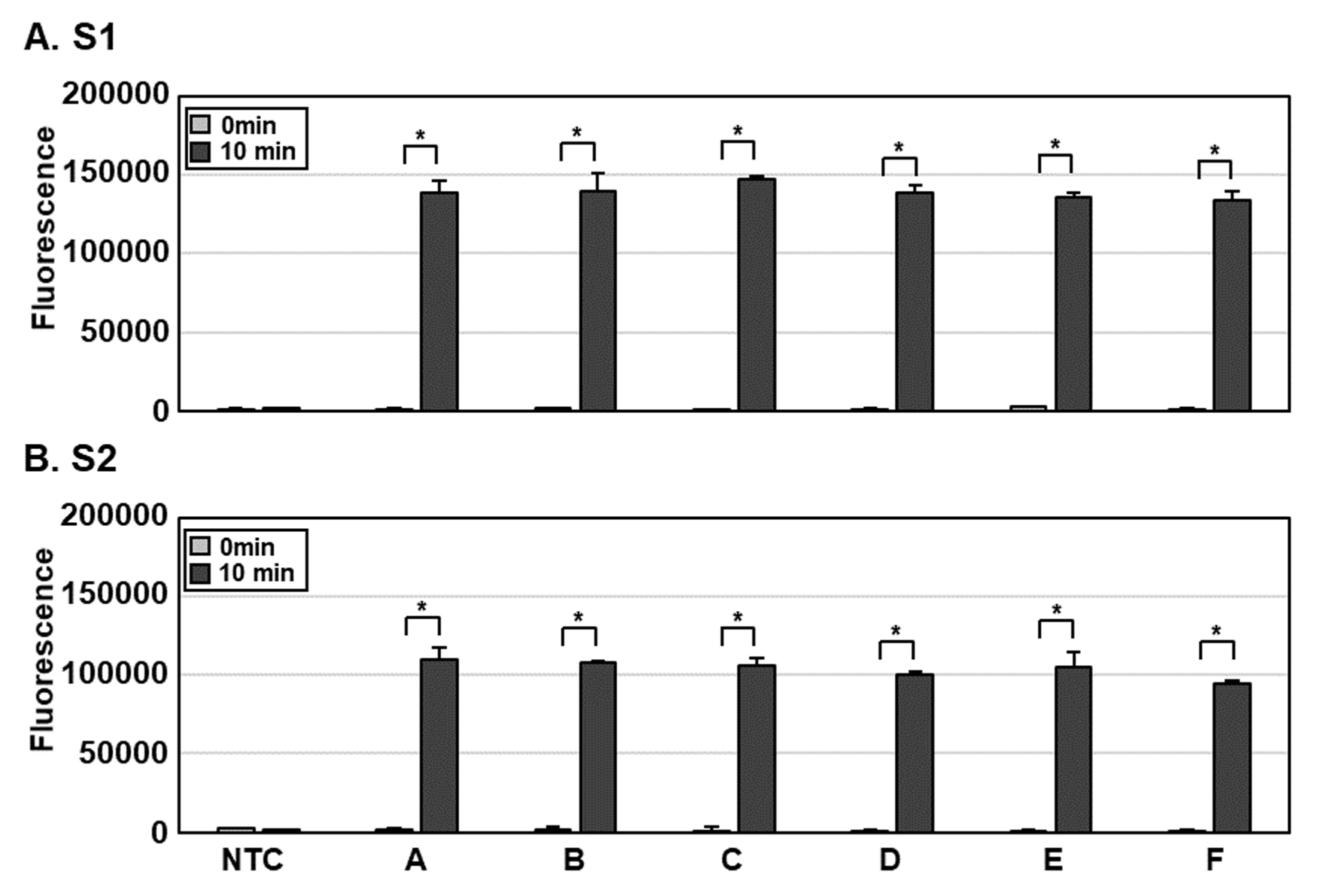

Supplement: S3 Fig — The in vitro-transcribed RNA fragments of S gene from SFTSV genotypes A, B, C, D, E and F were amplified via RT-RPA with a primer set specific for the SFTSV S gene. RT-RPA amplicons were detected using SFTSV DETECTR combined with fluorescence assay or lateral flow using gRNAs (A) S1 and (B) S2 corresponding to the S genes of all SFTSV genotypes. Fluorescence saturation occurred within 10 min. Lateral flow assay results were evaluated 2 min after the strip was reacted with the sample. Values are presented as means ± s.d. (error bars) (n = 3 replicates; * p < 0.05 between samples, two-samples t-test). NTC, no template control. (TIF) [file pntd.0010666.s003.tif]

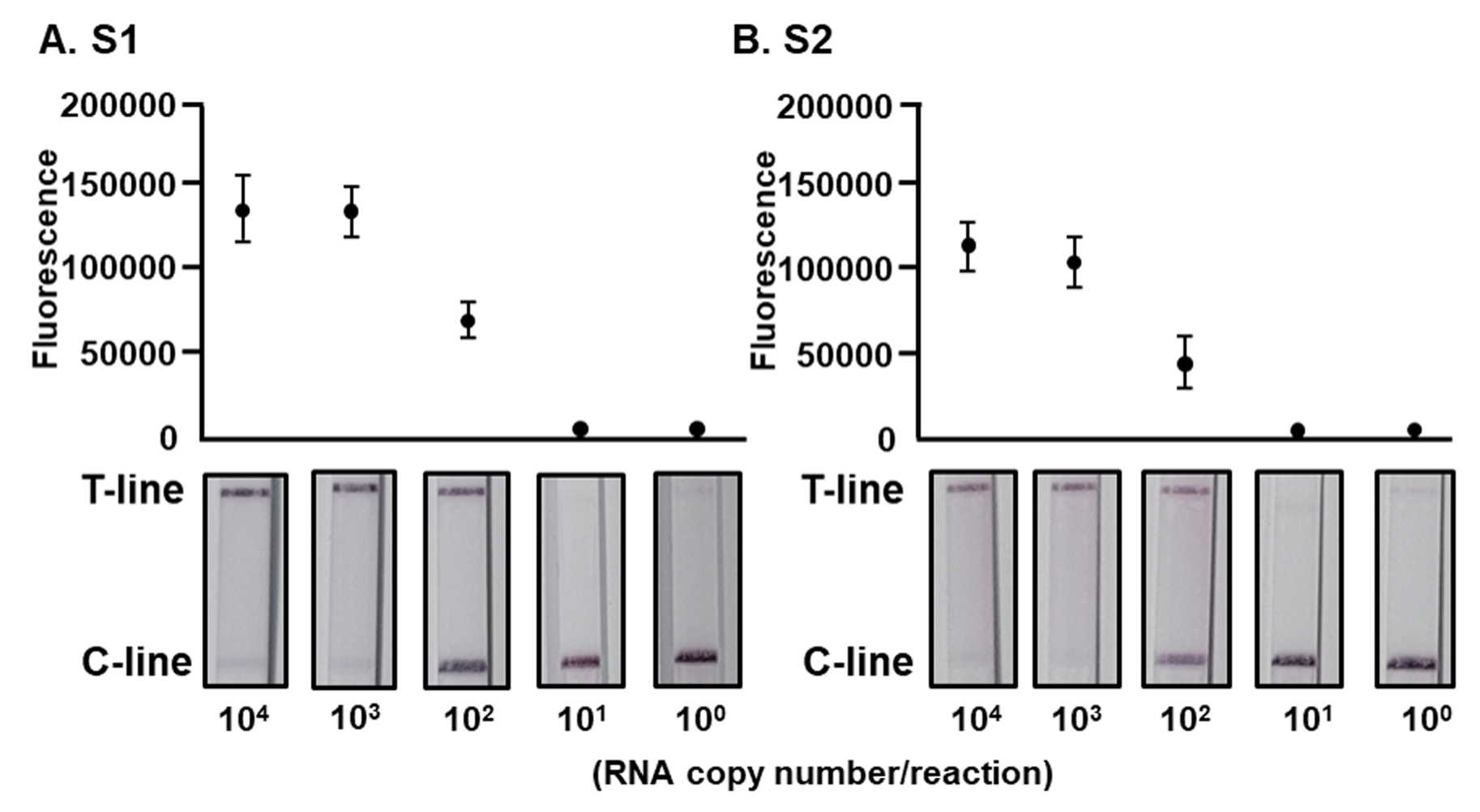

Supplement: S4 Fig — Different copy numbers of in vitro-transcribed RNA fragments of SFTSV S gene were amplified via RT-RPA with a primer set specific for the SFTSV S gene. RT-RPA amplicons were detected using SFTSV DETECTR combined with fluorescence assay or lateral flow using gRNAs (A) S1 and (B) S2 corresponding to the S genes of all SFTSV genotypes. The fluorescence was measured at 10 min. Lateral flow assay results were evaluated 2 min after the strip was reacted with the sample. Values are presented as means ± s.d. (error bars) (n = 3 replicates). C-line, control line; T-line, test line. (TIF) [file pntd.0010666.s004.tif]
