# Supplementary material for: A CRISPR-Cas12a-based diagnostic method for multiple genotypes of severe fever with thrombocytopenia syndrome virus
Source: PLoS Negl Trop Dis. 2022 Aug 2;16(8):e0010666. doi: 10.1371/journal.pntd.0010666 (PMC9345333; doi:10.1371/journal.pntd.0010666)
Supplement: S1 Table — (DOCX) [file pntd.0010666.s005.docx]

| **Genotype** | **Sequence** | |
| --- | --- | --- |
| SFTSV A | F’ | 5’-TAATACGACTCACTATAGGGAGAAAGTCTAAGTCTTCGCTTCTCTATGGC-3’ |
|  | R’ | 5’-GGGGACCACTTTGTACAAGAAAGCTGGCTATGTTCTTCTCCATCAAGAAC-3’ |
| SFTSV B | F’ | 5’-TAATACGACTCACTATAGGGAGAAAGTCTGAGCCTTCGCTTCTCTATGGC-3’ |
|  | R’ | 5‘-GGGGACCACTTTGTACAAGAAAGCTGGCTATGTTCTTCTCCATCAAGAAC-3’ |
| SFTSV C | F’ | 5‘-TAATACGACTCACTATAGGGAGAAAGTCTAAGTCTCCGCTTCTCTATGGC-3’ |
|  | R’ | 5’-GGGGACCACTTTGTACAAGAAAGCTGGCTATGTTCTTCTCCATCAAGAAT-3’ |
| SFTSV D | F’ | 5’-TAATACGACTCACTATAGGGAGAAAGTCTAAGCCTTCGCTTCTCAATGGC-3’ |
|  | R’ | 5’-GGGGACCACTTTGTACAAGAAAGCTGGCTATGTTCTTCTCCATCAAGAAC-3’ |
| SFTSV E | F’ | 5’-TAATACGACTCACTATAGGGAGAAAGTCTAAGCCTTCGCTTCTCTATGGC-3’ |
|  | R’ | 5’-GGGGACCACTTTGTACAAGAAAGCTGGCTATGTTCTTCTCCATCAAGAAC-3’ |
| SFTSV F | F’ | 5’-TAATACGACTCACTATAGGGAGAAAGTCTAAGCCTTCGCTTCTCTATGGC-3’ |
|  | R’ | 5’-GGGGACCACTTTGTACAAGAAAGCTGGCTATGTTCTTCTCCATCAAGAAC-3’ |

**S1 Table. PCR primers for IVT**
